# A Comparison of Online Versus Offline Gambling Harm in Portuguese Pathological Gamblers: An Empirical Study

**DOI:** 10.1007/s11469-017-9846-8

**Published:** 2017-11-29

**Authors:** Pedro Hubert, Mark D. Griffiths

**Affiliations:** 1 IAJ (Gambling Support Institute), Rua Sacadura Cabral 65, 1495-207 Lisbon, Portugal; 2 Psychology Department, Nottingham Trent University, 50 Shakespeare Street, Nottingham, NG1 4FQ UK

**Keywords:** Problem gambling, Gambling disorder, Online gambling, Gambling in Portugal, Gambling situational characteristics

## Abstract

Over the past decade, gambling has become a very popular activity across Europe including the growth of Internet gambling. Portugal is one of the few European countries where little research has been carried out. Given the lack of studies, a Portuguese sample (*N* = 1,599) was surveyed concerning their online and offline gambling habits. More specifically, the aim of this study was to identify and compare from the total sample, online pathological gamblers (PGON) (*n* = 171) and offline pathological gamblers’ (PGOF) (*n* = 171) characteristics, and eventual risk factors for the development of problem gambling. Results demonstrated that PGON had different profiles compared to PGOF, although there were also similarities. Situational characteristics were much more significant for PGON than PGOF (e.g., availability, accessibility, affordability), but PGOF had higher scores than PGON on factors concerning individual characteristics (e.g., intensity of feelings while gambling, depression, suicidal ideation, etc.). Findings also showed differences concerning attitudes toward responsible gambling measures. The fact that situational characteristics are more attractive to online gamblers confirms differences between PGON and PGOF and suggests that this preferred attractiveness may enhance problem gambling potential. Further research is needed to better understand the interaction between Internet situational characteristics and the individual characteristics of gamblers, as well as the profile of the growing population of gamblers that uses both online and offline modes to gamble.

Gambling has become an increasingly popular leisure activity throughout Europe (Calado and Griffiths 2016). New technologies have significantly facilitated this growth in the form of remote gambling including Internet gambling, mobile phone gambling, and interactive television gambling (Kuss and Griffiths 2012). Research examining Internet gambling suggests it may be more likely to contribute to problem gambling than gambling in offline environments among vulnerable individuals such as problem gamblers (Griffiths et al. 2009; Kuss and Griffiths 2012). There have been claims that increasing gambling opportunities via remote gambling have resulted in an increasing number of gamblers and that the problem is likely to get worse (Barrault and Varescon 2012). Identifying the specific characteristics of online and offline problem gamblers in specific cultures and/or jurisdictions may help in the development of culturally relevant prevention and treatment programs for gambling-related harm. Furthermore, variables such as cultural values and beliefs, the process of acculturation, and the influence of culturally determined help-seeking behaviors also need to be examined in relation to the role they could play in the initiation of and maintenance of gambling (Raylu and Oei 2004).

As Internet gambling has become more widespread, some studies have claimed that Internet gambling may be more problematic for vulnerable individuals because of its situational and structural properties such as availability, accessibility, affordability, anonymity, and convenience (McCormack and Griffiths 2013). Studies have also shown that gamblers themselves believe online gambling is more addictive than offline gambling and that online gambling will exacerbate gambling problems in society (e.g., McCormack and Griffiths 2012). Researchers have also attempted to establish whether offline problem gamblers differ from online problem gamblers (Griffiths 2010) and whether online gambling is more dangerous than offline gambling (Shaffer 2009). Despite the many European studies that have reported data on various aspects of gambling, data concerning online and offline gambling addiction in Portugal is almost non-existent. Based on the few studies carried out, the prevalence of gambling and problem gambling in Portugal appears to be similar to the corresponding figures in other European countries (Calado and Griffiths 2016). Gambling in Portugal has traditionally been confined to offline social gambling (e.g., *EuroMillions* and other lottery games, large land-based casinos, etc.). However, more recently, online betting has become more popular. In 2015, new gambling legislation was introduced regulating the industry in different areas both online and offline.

According to Lopes (2010), the South Oaks Gambling Screen (SOGS; Lesieur and Blume 1987) allows an assessment of the degree of pathology in relation to the gambling behavior on a continuum of severity of existing problems. Making use of the SOGS in Portugal, Lopes (2010) carried out a national representative survey (*N* = 3850) and reported that 48% of the general population gambled at least once in 2009. Problem gamblers were more likely to be male (71% men; 21% women), while at-risk gamblers were 52% male and 48% female. Also using the SOGS, Balsa (2012) reported a probable pathological gambling rate of 0.3% in a representative survey (*N* = 6817). There were five times as many male pathological gamblers as there were females. Balsa (2012) reported that the Portuguese population gambled on the following activities (listed in descending order): *Euromillions* lottery (61.6%), totobola/lotto (31.7%), scratchcards (24.6%), lotteries (18.3%), land-based casinos and bingo (6.5%), sports betting (5.4%), card games among friends or acquaintances for money (5.3%), skill games for money offline such as as snooker, pool, golf, etc. (3.6%), and betting between friends or acquaintances for money (3.2%).

Using the SOGS questionnaire, the Serviço de Intervenção nos Comportamentos Aditivos e nas Dependências (2017) reported that 46.2% of Portuguese adult population gambled and did not have any problems with gambling, 1.2% showed some problems with gambling, and 0.6% are probable pathological gamblers. Compared to 2012, the prevalence of problem gambling rose from 0.3 to 1.2%, and the prevalence of pathological gambling rose from 0.3 to 0.6%. According to the Serviço de Regulação e Inspeção de Jogos (2017), Portugal had, from June 2016 to June 2017, more than 587,000 players accessing online gambling platforms and 11,500 voluntary self-excluders. The only other studies on gambling using Portuguese are those examining adolescent gambling using focus groups (i.e., Calado et al. 2014) and surveys (Calado et al. 2016, 2017) showing that a small minority of Portuguese adolescents are problem gamblers when assessed using the Portuguese validation of the multiple response juvenile version of the DSM-IV criteria (DSM-IV-MR-J; Calado et al. 2016).

It has been reported that if governments want to promote a culture of sustainable and responsible gambling, then they need an understanding of the potential relationship between Internet gambling and problem gambling (Wood and Williams 2007). Research to date has shown there are differences and similarities between online and offline gamblers. Understanding the chain of progression from recreational gambling to pathological gambling is vital in understanding its pathogenesis (Odlaug et al. 2011). It may be the case that the online medium is more dangerous for problem and pathological gamblers rather than the medium itself being the main issue of concern (Kuss and Griffiths 2012). Very few empirical studies have directly compared Internet gambling with land-based gambling using representative samples. Using data from the British Gambling Prevalence Survey (*N* = 7756), Wardle et al. (2011) reported that mixed-mode gamblers (i.e., those that gambled both online and offline on a variety of different gambling games) showed the highest involvement as well as the highest percentage of problem gambling. More specifically, the highest prevalence rates of problem gambling were among mixed-mode gamblers who gambled on different activities (4.3%), followed by mixed-mode gamblers who gambled on the same activities (2.4%), those who only gambled offline (0.9%), and those who only gambled online (0%).

Many different types of factors contribute to the acquisition, development, and maintenance of problem gambling. Individual characteristics include (i) sociodemographic variables such as age, gender, socioeconomic status, early childhood experiences, and influence of parental gambling; (ii) personality factors such as motivation to gamble, arousal and sensation seeking, impulsivity, drive reduction, mood regulation, and dissociation; and (iii) preferred gambling activity and cognitive variables such as illusion of control, interpretative biases (e.g., attribution biases, gambler’s fallacy), and illusory control over luck and chance (Toneatto and Nguyen 2007). Data are emerging that pathological gambling is a disorder that rarely occurs in isolation, and that is often related to other psychiatric conditions such as substance use disorders, mood disorders, anxiety disorders, other disorders as comorbidities (Petry 2005).

Factors external to the gambler include situational characteristics. These refer to the physical and social environments individual find themselves in while gambling either online or offline (Kuss and Griffiths 2012). Situational factors that affect gambling behavior include availability, accessibility, exposure and other locations, and contextual factors as alcohol, tobacco, marketing, and advertising (Abbott 2007). Such factors may be of particular importance in relation to online gambling. For instance, the main reasons why people prefer online over offline gambling include the relative convenience, comfort and ease of Internet gambling, an aversion to the atmosphere and clientele of land-based venues, a preference for the pace and nature of online game-play, and the potential for higher wins and lower overall expenditure when gambling online (Griffiths and Barnes 2008; McCormack and Griffiths 2012; Wood et al. 2007). The study of online and digital behavior is of particular importance and the present study attempts to better understand Internet gambling and pathological gambling.

Given the relative lack of research examining the differences between online and offline gambling coupled with the fact that data concerning Portuguese gambling is scarce, the primary aim of the present study was to compare online and offline gamblers in relation to individual and situational characteristics. It was hypothesized that situational characteristics of Internet problem gambling (i.e. availability, accessibility, affordability, anonymity, convenience) would have a significant impact on vulnerable individuals that gamble online (i.e., PGON), resulting in significantly higher attractiveness scores in the PGON group rather than PGOF group. This exploratory study examined differences in motivations, attitudes, consequences, and sociodemographics between online pathological gamblers (PGON) and offline pathological gamblers (PGOF) in a Portuguese sample. The main focus was essentially centered on attractiveness of situational characteristics, responsible gambling measures, and individual factors such as age, gender, sensation seeking, and psychosocial symptoms in a Portuguese sample.

## Method

### Participants

The total sample of this study comprised 1599 participants (1008 males and 591 females) aged from 16 to 83 years (M = 31.7 years; SD = 12.0 years). The sample was divided in three groups according to SOGS scores: (1) no problem gambler or recreational gambler (*n* = 481; 30.1% of sample), (2) some problems with gambling or at-risk gambler (*n* = 776; 48.5%), and (3) probable pathological gambler or pathological gambler (*n* = 342; 21.4%). Each of the three groups was then sub-divided according to their main gambling mode: (a) online gamblers (*n* = 959) or (b) offline gamblers (*n* = 640) resulting in six groups: (1a) online recreational gamblers (*n* = 306); (2a) online at-risk gamblers (*n* = 482), (3a) online pathological gamblers (PGON) (*n* = 171), (1b) offline recreational gamblers (*n* = 175), (2b) offline at-risk gamblers (*n* = 294), and (3b) offline pathological gamblers (PGOF) (*n* = 171). All participants below the age of 16 years, non-Portuguese nationalities, as well as those who did not live in Portugal were not included in the analysis.

### Procedure

An advertisement to participate in the study was made primarily through television, radio, and press announcements, giving to gamblers the possibility to assess their degree of involvement in gambling (provided by SOGS). The study was open to all participants who wanted to know their degree of dependence to gambling. Confidentiality and anonymity were guaranteed to all participants. A website was designed by the research team to host two identical questionnaires except for the fact that one set of questions related to online gambling while the other related to offline gambling. Participants completed one of the questionnaires depending upon which mode of gambling they primarily gambled upon. The amount of time participants took to complete the questionnaires was approximately 15–20 min. Online data collection was chosen as there is much evidence that when properly conducted, online surveys generate data that are just as valid (if not more so) than data collected using traditional forms (Wood and Williams 2007). As exclusion criteria, the IP addresses were coded to ensure no person filled out the questionnaire more than once, but no duplicate answers were found among the completed surveys.

### Design

The study was descriptive, exploratory, quantitative, and comparative. The design was chosen in order to obtain maximum representativeness of at-risk and pathological gamblers in the Portuguese population.

### Measures

For the present study, two instruments were used and were made available online. The first instrument comprised a self-devised questionnaire (available on request from the first author) and included 15 basic questions (including demographic variables such as age, gender, nationality, country of residence), followed by 41 questions directed towards Portuguese gambling culture (i.e., history of gambling behaviors, types of game played, money and time spent, motivations to gamble, consequences of gambling, etc.) but based on international surveys such as those produced by the British Gambling Commission (Wardle et al. 2007) and the New Zealand Helpline (2010). In the second part of this first instrument, most questions had “yes” or “no” responses (31 questions), and all mean scores (except one question), were based on a Likert scales (10 questions), and comprised three dimensions: (i) individual characteristics and psychosocial symptoms concerning offline/online gambling (e.g., depression, anxiety, suicide attempts, other dependencies such as tobacco, substances, and alcohol), (ii) gambling behaviors and attractiveness of online gambling (situational characteristics, time and amount of money spent in gambling, chasing, convenience, availability), and (iii) responsible gambling measures (e.g., self-exclusion, helplines).

The second instrument used to assess problem gambling was the Portuguese version of SOGS (Lesieur and Blume 1987). This instrument was adapted for the Portuguese population by Lopes (2010). The SOGS evaluates the degree of pathology of gambling behaviors comprising 20 dichotomous questions (yes/no). The scores range from 0 to 20, according to the number of positive responses. The classification of gamblers ranges from “no problems with gambling” (0 out of 20), “some problems with gambling” (1–4 out of 20), to “probable pathological gamblers” (5 or more out of 20). Although the new official classification in the DSM-5 (APA 2013) now terms pathological gambling as gambling disorder, the present study defined groups based on SOGS classifications. According to the psychological literature, the SOGS better fits the Portuguese culture (Lopes 2008), and is preferred by Portuguese population compared with other screens (Lopes 2010).

### Ethics

The study procedures were carried out in accordance with the Declaration of Helsinki. The research team’s University Ethics Committee approved the study. All participants were informed about the study and all provided informed consent.

### Statistical Analysis

Although there were six different groups in this exploratory study, the focus of the present paper is the direct comparison of online pathological gamblers (PGON) and offline pathological gamblers (PGOF). The analysis sought to identify characteristics and risk factors for problem gambling among Portuguese citizens. To compare the two groups, Kolmogorov–Smirnov analysis of normality was conducted on all dependent variables of the study in the two instruments (i.e., the self-devised questionnaire and SOGS). Following this analysis, comparative *t* test analyses in normative variables and Mann-Whitney tests in non-normative variables were carried out. In order to compare groups on nominal variables, chi-square tests were carried out. ANOVAs were also carried out to compare and describe differences between all the groups obtained with SOGS (groups 1, 2, and 3 of the study). Due to the large amount of data analyzed, the present paper mainly reports the results of variables where statistically significant differences were found between the groups. Findings are presented according to three dimensions: (i) individual characteristics, (ii) attractiveness of online gambling, and (iii) attitudes toward responsible gambling measures.

## Results

### Gambling Behavioral and Individual Characteristics on Offline/Online Gambling

There was a significant difference in age between PGOF (M = 39.75; SD = 12,544) and PGON (M = 31.05; SD = 11.24) (*t*(340) = 6757; *p* < 0.001). Almost half of PGON (44.7%) were aged from 16 to 20 years (24.4%) and one-fifth from 21 to 25 years (20.3%). More than 70% of online and offline pathological gamblers in the sample were male. PGOF were more likely to have jobs (*χ* ^*2*^(1) *=* 5.420; *p* < 0.020), have children (*χ* ^*2*^(1) = 8.477; *p* < 0.004), and have a stable relationship (*t*(244) = − 2.6465; *p* < 0.014) (see Table 1). There were more male PGON (79.3%; 20.7% females) than male PGOF (72.6%; 27.4%) but this did not reach statistical significance. Results also showed that more than 80% of PGON and 75% of PGOF had more than 12 years of education and/or 3 years of university education, and that approximately 60% of the household income was between 15,000 and 60,000 Euros for both groups. Results also showed that PGON and PGOF were equally likely to be engaged in a relationship (71.3 vs. 71.6%), equally satisfied with their relationships (means = 5.11 vs. 5.81), and equally likely to be living in urban or suburban areas (89.7 vs. 89.9%).

**Table 1:** Sociodemographics of online and offline gamblers and comparison between pathological online and offline gamblers

		Offline gambler’s characterization					Online gambler’s characterization					Comparison: PGOF/PGON					
		Recreational	At-risk abusive	Pathologic	Total	Sig.	Recreational	At-risk abusive	Pathologic	Total	Sig.	Offline	Online	Total	*χ* ^2^	d.f.	Sig.
Gender	Female	93	117	46	256	0.001*	156	144	35	335	0.001*	46	35	81	2.053	1	0.152
		36.3%	45.7%	18.0%	100.0%		46.6%	43.0%	10.4%	100.0%		27.4%	20.7%	24.0%			
	Male	79	172	122	373		147	333	134	614		122	134	256			
		21.2%	46.1%	32.7%	100.0%		23.9%	54.2%	21.8%	100.0%		72.6%	79.3%	76.0%			
Age	Mean	34.74	33.62	39.75	35.58	0.001*	29.84	27.94	31.05	29.11	0.001*	39.75	31.05	35.40	6.757	340	0.001*
	SD	12.80	12.38	12.54	12.78		11.60	9.92	11.27	10.78		12.54	11.23	12.67	^a^		
Education	Up to 9 years of education	26	25	40	91	0.001*	47	48	31	126	0.003*	40	31	71	2.348	2	0.309
		28.6%	27.5%	44.0%	100.0%		37.3%	38.1%	24.6%	100.0%		23.7%	18.5%	21.1%			
	12 years education	58	108	60	226		106	221	72	399		60	72	132			
		25.7%	47.8%	26.5%	100.0%		26.6%	55.4%	18.0%	100.0%		35.5%	42.9%	39.2%			
	License	83	157	69	309		142	200	65	407		69	65	134			
		26.9%	50.8%	22.3%	100.0%		34.9%	49.1%	16.0%	100.0%		40.8%	38.7%	39.8%			
Job	Yes	118	192	129	439	0.056	163	259	111	533	0.030*	129	111	240	5.420	1	0.020*
		26.9%	43.7%	29.4%	100.0%		30.6%	48.6%	20.8%	100.0%		76.8%	65.3%	71.0%			
	No	54	98	39	191		140	217	59	416		39	59	98			
		28.3%	51.3%	20.4%	100.0%		33.7%	52.2%	14.2%	100.0%		23.2%	34.7%	29.0%			
Household income in Euros	Up to 15.000	60	98	49	207	0.752	116	178	56	350	0.327	49	56	105	1.245	3	0.742
		29.0%	47.3%	23.7%	100.0%		33.1%	50.9%	16.0%	100.0%		29.0%	33.7%	31.3%			
	15.001 to 40.000	41	70	49	160		75	124	47	246		49	47	96			
		25.6%	43.8%	30.6%	100.0%		30.5%	50.4%	19.1%	100.0%		29.0%	28.3%	28.7%			
	40.001 to 60.0000	59	91	56	206		68	133	52	253		56	52	108			
		28.6%	44.2%	27.2%	100.0%		26.9%	52.6%	20.6%	100.0%		33.1%	31.3%	32.2%			
	> 60.000	12	28	15	55		27	28	11	66		15	11	26			
		21.8%	50.9%	27.3%	100.0%		40.9%	42.4%	16.7%	100.0%		8.9%	6.6%	7.8%			
Engaged relationship	Relation	129	206	122	457	0.652	186	281	121	588	0.016*	122	121	243	0.003	1	0.959
		28.2%	45.1%	26.7%	100.0%		31.6%	47.8%	20.6%	100.0%		71.3%	71.6%	71.5%			
	No relation	43	83	49	175		115	194	48	357		49	48	97			
		24.6%	47.4%	28.0%	100.0%		32.2%	54.3%	13.4%	100.0%		28.7%	28.4%	28.5%			
Stability in relationship	Mean	6.33	6.08	5.30	5.94	0.001*	6.29	6.21	5.81	6.15	0.003*	5.30	5.81	5.55	−2.465	244	0.014*
	SD	1.14	1.40	1.77	1.50		1.11	1.28	1.46	1.28		1.77	1.46	1.64	^a^		
Satisfaction with relationship		6.25	5.97	5.11	5.81	0.001*	6.22	6.04	5.52	5.99	0.001*	5.11	5.52	5.31	−1.781	251	0.076
		1.23	1.48	1.94	1.62		1.15	1.38	1.66	1.40		1.94	1.66	1.81	^a^		
Children	Yes	57	83	70	210		73	96	45	214		70	45	115	8.477	1	0.004*
		32.8%	28.7%	41.7%	33.3%	0.018*	24.3%	20.2%	26.6%	22.6%	0.158	41.7%	26.6%	34.1%			
	No	117	206	98	421		227	380	124	731		98	124	222			
		67.2%	71.3%	58.3%	66.7%		75.7%	79.8%	73.4%	77.4%		58.3%	73.4%	65.9%			
Place of residence	Rural	31	35	17	83	0.087	50	81	17	148	0.251	17	17	34	0.534	2	0.766
		37.3%	42.2%	20.5%	100.0%		33.8%	54.7%	11.5%	100.0%		10.3%	10.2%	10.2%			
	Urban	109	216	126	451		204	315	123	642		126	123	249			
		24.2%	47.9%	27.9%	100.0%		31.8%	49.1%	19.2%	100.0%		76.4%	73.7%	75.0%			
	Suburban	31	43	22	96		46	82	27	155		22	27	49			
		32.3%	44.8%	22.9%	100.0%		29.7%	52.9%	17.4%	100.0%		13.3%	16.2%	14.8%			

*PGOF* pathological gamblers offline, *PGON* pathological gamblers online

*p ≤ 0.05,**p ≤ 0.10

^a^Independent-samples *t* test

Results showed that compared to PGOF, PGON gambled more hours per day (4.61 vs. 4.37; *U* = 11,632.5; *p* < 0.628), per weekend day (7.85 vs. 7.13; *U* = 9908.5; *p* < 0.515), and more days per week (5.14 vs. 3.33; *U* = 6973.5; *p* < 0.001). They also reported that they had increased the amount of time and money spent during last year (46.5 vs. 55.6%; *χ* ^*2*^(1) = 2.634; *p* < 0.105), although there was no statistically significant differences. Results showed an increased progression in quantity of time and money spent on the recreational/at-risk/pathological continuum in both online and offline groups. Although not statistically significant, results showed that betting with money was very attractive for both groups (PGON = 76.6% vs. PGOF = 83.6%),

Results showed that pathological gamblers and at-risk gamblers had very similar results in relation to the device and place from where they gambled most. The home computer was the most used hardware for gambling (69.6 vs. 65.4%) followed by laptops in several places (63.2 vs. 64.9%), mobile telephone (19.9 vs. 16.6%), and work computer (17 vs. 9.5%). The mean age that PGON started to interact with computers was statistically different from PGOF (16.20; SD = 9.80 years vs. 22.64; SD = 12.66 years old; *t*(524) = 5147; *p* < 0.001). Results also showed that PGON mostly gambled alone (62.7%) compared to 47% of online at-risk gamblers. PGOF also reported gambling mostly alone (64.5%) with a small minority gambling alone or with others (18.9%).

As gambling intensity increased, gamblers bet more money on the different gambling types (both online and offline). Results showed there were significant differences concerning the types of games on which both pathological groups mostly played. With the exception of the *EuroMillions* lottery game (that is the most played by both groups), lotteries, and scratchcards (that PGOF play slightly more than PGON), all other gambling types showed significant differences between the two groups of gamblers. The favorite gaming activities for PGON were poker (47.4%), sports betting (45.03%), online videogames such as MMORPGs or FPS games (31.58%), and social networking games on sites like *Facebook* (25.73%). The favorite gaming types for PGOF were slot machines (66.1%), casino card games (66.1%), *EuroMillions* lottery (57.3%), lotteries (21.1%), and scratchcards (18.1%). Results showed that 8.2% pathological gamblers exclusively bet online, 21.6% bet exclusively offline, and the remainder (70.2%) gambled both online and offline.

The results concerning the intensity of feelings in both PGON and PGOF were significantly different with higher means for PGOF on items such as experiencing euphoria (means = 4.04; SD = 1.046 vs. 3.71; SD = 1.037; *t*(334) = 2866; *p* < 0.001), escape (means = 3.98; SD = 11.154 vs. 3.53; SD = 1.221; t(331) = 3.401; *p* < 0.001), losing time quickly (means = 4.34; SD = 1.068 vs. 4.08; SD = 1.047; *t*(332) = 2.23; *p* < 0.001), having suicidal thoughts (33.3 vs. 17%; *χ* ^*2*^(1) = 9.49, *p* < 0.002), and very similar figures concerning suicide attempts (7.6 vs. 8.2%; *χ* ^*2*^(1) = 0.040; *p* < 0.841).

### Gambling Behavior and the Attractiveness of Online Gambling (Situational Characteristics)

Results demonstrated many significant differences between PGON and PGOF in relation to the advantages of using the Internet to gamble (see Table 2). Although there was no difference between the two groups in relation to feeling a connection with other people (*p* < 0.221), PGOF were significantly more likely than PGON to play to win money (means = 4.07; SD = 1.16 vs. 3.67; SD = 1.37; *U* = 11,193.5; *p* < 0.007). There were always higher means for PGON vs. PGOF on all other gambling motivations: accessibility (4.37 vs. 2.32; *U* = 3218; *p* < 0.001), round the clock availability (4.37 vs. 2.74; *U* = 4411.5; *p* < 0.001), convenience (4.14 vs. 3.19, *U* = 7450; *p* < 0.001), fun (4.10 vs. 3.80; *U* = 11,224.5; *p* < 0.037), diversity of games (3.47 vs. 3.01; *U* = 10,265.5; *p* < 0.004), easy to end the game (3.13 vs. 2.51; *U* = 8585.5; *p* < 0.001), anonymity/privacy (3.08 vs. 2.45, *U* = 9236.5; *p* < 0.001), diversity of sites (3.05 vs. 2.35; U = 8823; *p* < 0.001), and prevention/protection (3.0 vs. 2.42; *U* = 8952; *p* < 0.001).

**Table 2:** Responses to the question “What attracts you most about offline/online gambling?”

		Offline gambler’s characterization				Online gambler’s characterization				Comparison: PGOF/PGON				
		Recreational	At-risk abusive	Pathologic	Sig.	Recreational	At-risk abusive	Pathologic	Sig.	Offline	Online	(U)^a^		Sig.
Anonymity	Mean	2.98	3.13	2.45	0.001*	2.61	2.71	3.08	0.004*	2.45	3.08	9236.5		0.001*
Privacy	SD	1.68	1.52	1.40		1.45	1.49	1.51		1.40	1.51			
Convenience	Mean	2.69	3.22	3.19	0.001*	3.68	3.79	4.14	0.001*	3.19	4.14	7450		0.001*
	SD	1.40	1.35	1.32		1.31	1.25	1.09		1.32	1.09			
Access (fast and easy)	Mean	1.69	1.91	2.32	0.001*	3.88	4.04	4.37	0.001*	2.32	4.37	3218		0.001*
	SD	1.12	1.23	1.37		1.21	1.10	0.87		1.37	0.87			
Availability (24 h/day)	Mean	2.12	2.36	1.35	0.001*	3.76	4.05	4.37	0.001*	2.74	4.37	4411.5		0.001*
	SD	1.31	1.35	1.38		1.31	1.6	0.98		1.38	0.98			
Diversity of games	Mean	2.48	2.77	3.01	0.005*	3.58	3.45	3.47	0.470	3.01	3.47	10,265.5		0.004*
	SD	1.41	1.46	1.44		1.36	1.39	1.37		1.44	1.37			
Diversity of sites/places	Mean	2.13	2.34	2.35	0.163	2.92	3.00	3.05	0.648	2.35	3.05	8823		0.001*
Sites	SD	1.30	1.31	1.32		1.43	1.43	1.37		1.32	1.37			
Connection to others	Mean	2.23	2.45	2.41	0.201	2.67	2.77	2.63	0.436	2.41	2.63	11,174		0.221
	SD	1.40	1.44	1.31		1.41	1.43	1.45		1.31	1.45			
Easy to end	Mean	2.50	2.63	2.51	0.502	3.05	3.19	3.13	0.421	2.51	3.13	8585.5		0.001*
	SD	1.50	1.40	1.33		1.44	1.39	1.41		1.33	1.41			
To win money	Mean	2.05	2.82	4.07	0.001*	1.90	2.63	3.67	0.001*	4.07	3.67	11,193.5		0.007*
	SD	1.43	1.53	1.16		1.31	1.49	1.37		1.16	1.37			
Fun	Mean	3.72	4.02	3.80	.058**	4.38	4.34	4.10	0.001*	3.80	4.10	11,224.5		0.037*
	SD	1.47	1.20	1.20		0.97	0.98	0.97		1.20	0.97			
Prevention and protection	Mean	2.24	2.39	2.42	.269	2.93	2.91	3.00	0.804	2.42	3.00	8952		0.001*
	SD	1.47	1.42	1.34		1.47	1.42	1.37		1.34	1.37			

*p ≤ 0.0

^a^Mann-Whitney

### Responsible Gambling Measures

In relation to protection and prevention measures while gambling, PGON had significantly more confidence or trust in all measures than PGOF except for limit-setting measures (both time and money), and feeling protected from crime. For the PGON group, the most endorsed responsible gambling measures were privacy and protection (mean = 3.64; SD = 1.34 vs 2.91; SD = 1.43; *U* = 8661.5; *p* < 0.001), information on rules and probabilities (3.48; SD = 1.28 vs 2.80; SD = 1.33; *U* = 8320.5; *p* < 0.001), access to betting history (3.40; SD = 1.42 vs 2.51; SD = 1.36; *U* = 7718.5; *p* < 0.001), money and time controls (3.10; SD = 1.45 vs 2.99; SD = 1.47; *U* = 12,314.5; *p* < 0.494), self-exclusion options (3.06; SD = 1.45 vs 2.56; SD = 1.40; *U* = 9371.5; *p* < 0.003), and professional help (2.17; SD = 1.27 vs 2.63; SD = 1.35; *U* = 9391.5; *p* < 0.002). In general, the endorsement of responsible gambling measures increases across the online gambling group (i.e., recreational gamblers are least likely to endorse RG measures whereas the pathological gamblers are most likely). Among the offline gambling group, it is the at-risk gamblers that have typically higher endorsements of RG measures (see Table 3).

**Table 3:** Responses to the question “On a prevention level do you feel more protected/secure in which situation?”

		Offline gambler’s characterization				Online gambler’s characterization				Comparison: PGOF/PGON			
		Recreation	At-risk abusive	Pathologic	Sig.	Recreation	At-risk abusive	Pathologic	Sig.	Offline	Online	(U)^a^	Sig.
Money and time control	Mean	3.20	3.40	2.99	0.019*	2.98	3.26	3.10	0.052*	2.99	3.10	12,314.5	0.494
	SD	1.66	1.47	1.47		1.52	1.44	1.45		1.47	1.45		
Self-exclusion	Mean	2.17	2.40	2.56	0.034*	2.34	2.67	3.06	0.001*	2.56	3.06	9471.5	0.003*
	SD	1.41	1.40	1.40		1.41	1.39	1.45		1.40	1.45		
More information (rules, probability)	Mean	2.75	2.93	2.80	0.405	2.94	3.26	3.48	0.001*	2.80	3.48	8320.5	0.001*
	SD	1.48	1.50	1.33		1.41	1.30	1.28		1.33	1.28		
Historical information (bets, hands)	Mean	2.10	2.07	2.51	0.001*	2.50	2.99	3.40	0.001*	2.51	3.40	7718.5	0.001*
	SD	1.35	1.33	1.36		1.49	1.44	1.42		1.36	1.42		
Professional help	Mean	2.54	2.59	2.63	0.749	1.90	2.12	2.17	0.009*	2.63	2.17	9391.5	0.002*
	SD	1.49	1.45	1.35		1.22	1.24	1.27		1.35	1.27		
Privacy protection	Mean	3.02	3.02	2.91	0.715	2.99	3.30	3.64	0.001*	2.91	3.64	8661.5	0.001*
	SD	1.56	1.50	1.43		1.54	1.42	1.34		1.43	1.34		
Criminality protection	Mean	2.57	2.65	2.46	0.573	2.05	2.18	2.28	0.133	2.46	2.28	9353	0.495
	SD	1.68	1.57	1.43		1.12	1.13	1.14		1.43	1.14		

*p ≤ 0.05

^a^Mann-Whitney

## Discussion

The present study is the first to compare online and offline gambling among a Portuguese population. The study demonstrated evidence of several differences between PGOF and PGON in both individual and external dimensions (i.e., situational characteristics). However, these differences are not conclusive in confirming Internet gambling as being more harmful or more problematic than offline gambling, although they could be perceived in such a way. In this study, most online pathological gamblers were more likely to endorse statements related to situational characteristics influencing their gambling than individual characteristics. The combination of external and individual risk factors may create augmented risks for problem gambling. The introduction of the Internet to gambling activities may change some of the fundamental situational and structural characteristics and make them potentially more addictive and/or problematic for susceptible individuals (Griffiths and Barnes 2008). Based on the findings of the present study, situational characteristics are important factors regarding the attractiveness and potential harmfulness of gambling. Based on the results of the present study here in Portugal, the PGON are more susceptible to the situational characteristics of gambling. Consequently, there is an additional responsibility that gambling promoters, legislators, and the gambling regulators should implement at various levels (e.g., more preventive information, time/money limits, marketing, self-exclusion, etc.) particularly focused on youth gamblers.

PGON also had higher mean scores on individual characteristics such as motivations to gamble (e.g., betting for money, daytime plus nighttime gambling opportunity, etc.) and consequences (e.g., increased amount of time and money spent, etc.). The fact that PGON were much younger than PGOF (mean 31.05 vs. 39.75 years) may indicate strong risk factors that are inherent to youth—such as immaturity, identity construction, and poor decision-making and problem-solving, and poor impulse control, that may contribute to the development of pathological gambling. Previous research has emphasized that adolescents do not appear to distinguish between the concepts of probability, fate, luck, and chance, and that young problem gamblers have more faith in their ability to manipulate chance and “beat the system” (Froberg 2006; Griffiths 2011; Moore and Ohtsuka 1999). The results demonstrated that almost half of PGON were between 16 and 20 years old, and the age and is a cause for concern. Public and private services (i.e., those involved in treatment, prevention, research) should redirect their strategies to this new gambling accessibility mode and young age (such as online treatments and tracking systems targeted at this “new” online gambling population). One major consequence of young PGON appears to be that, although they appear to have a shorter duration before they become problem gamblers (i.e., 30 years old compared to 40 years for PGOF) and fewer suicidal thoughts, they have less control over their impulsiveness and coping with frustration, which may explain why there is the same level of PGON suicide attempts as PGOF. Dedicated health professionals, telephone helplines, and other strategies facilitating urgent intervention should be introduced in Portugal for these specific situations.

Previous research has shown that adolescent at-risk/problem Internet gamblers appear to be specifically associated with non-peer involvement, heavy alcohol use, and poor academic functioning (Potenza et al. 2011). Given that PGON are younger, this will influence various other parameters—such as being unemployed, having fewer children, and having less money—which were significantly different to those gambling offline. There are even fewer female pathological online gamblers than those gambling offline. This lower number of female PGON is difficult to explain (see Table 1). However, their proportion is increasing in the 16–20-year-old range, possibly due to the early exposure of Internet technologies of these new generations, the availability of gambling within social networks, and targeted marketing. Gender differences in gambling behaviors have been reported, both with respect to types of problem gambling for women as compared to men, as well as regarding patterns for the development of gambling problems (Potenza 2009) and males would be significantly more likely to be problem Internet gamblers than females (Griffiths and Barnes 2008). PGOF gambled fewer hours a week compared to the PGON group, and believed that less gambling time was more suitable/balanced. PGOF gambled in shorter periods, but more intensively and in an uncontrolled way that increased their negative consequences. This was coupled with the other results comparing PGOF and PGON. The results showed that offline pathological gamblers were winning and spending more money; had more intense feelings of excitement and evasion, had more alcohol, drug, and tobacco-related problems; had more diagnoses of depression, anxiety, stress; and had higher ratings on their need to substitute gambling with work, sex or television than PGON, although these latter items did not have a statistically significant difference. Pathological gamblers, most probably, already had some individual vulnerability regarding comorbidities such as other addictions. As their gambling problem develops their vulnerabilities (such as depression) may also get worse (i.e., a gambler who uses gambling as a strategy to overcome their unwanted mood states may develop gambling problems, and may also worsen their depression). These results may indicate the pathogenesis of longer problematic gambling careers and requires a different treatment approach (i.e., medication, inpatient treatment).

PGON exposure to gambling may be less intense and less prolonged in time and consequences because they are on average 10 years younger than PGOF. Given that in the present study vulnerable online gamblers acquired problem gambling 10 years earlier than offline gamblers, more help for this population is needed including online interventions and dedicated public youth departments, as well as strategic partnerships with schools, colleges, universities, and employment centers. The PGOF group demonstrated higher degrees of sensation seeking while gambling than PGON, including excitement/euphoria, evasion/escape, and disconnection while gambling. This might indicate the need to compensate for the negative consequences of a longer problem gambling career (such as the increased amount time and money spent, family conflicts, homeless situations, anxiety, depression, etc.) with higher involvement during gambling sessions, or could perhaps be a symptom of the loss of control associated with the longer time in developing problem gambling. Given that a high percentage of PGOF also gamble online, the search for these reinforcing experiences can be facilitated easily on the Internet, representing another risk factor in the development of problem gambling severity (i.e., as land-based gambling venues close, problem gamblers may go home and continue to gamble online).

The 24/7 accessibility of online gambling may be a way or possibility for offline pathological gamblers to continue their gambling. The limited data to date suggest that it is not the medium of gambling that is more problematic *per se* but that to vulnerable people such as problem gamblers, the Internet may be providing easily accessible “convenience” gambling that perhaps explains why problem gambling prevalence rates among online gamblers appear to be much higher than non-online gamblers (Griffiths and Auer 2011). However, this is still a cause for concern as it suggests that the Internet is a facilitating factor in the development of already vulnerable individuals (Griffiths and Barnes 2008).

The main situational dimension in this study was related to attractiveness. The question in the survey was: *What attracts you more about online or offline gambling?* With the exception of winning money, all variables had higher figures for PGON and all had statistically significant differences (see Table 2). These figures are most meaningful when we consider the individual characteristics of PGON concerning age, unemployment, impulsiveness, depression, anxiety, substance abuse, and sensation seeking, combined with the situational characteristics of the Internet. Individual characteristics of PGON and situational characteristics concerning online gambling mode may contribute an augmented risk factor due to the strong relationship that may be created (e.g., youth and impulsiveness combined with 24/7 availability). Other risk factors are associated with this emerging online population; the “always on” culture, unemployment, online peer pressure, online aggressive marketing, streaming (sport betting, videogames tournaments, and bets), and devices that allow access anywhere and on the move (e.g., smartphones, tablets, laptops, etc.).

With the exception of *EuroMillions*, games such as poker and live sport betting (which were more likely to be played by online pathological gamblers) have the following characteristics that may facilitate problematic use: rapid event frequency, high/fast money rewards, no spending limits, no ending, convenience, easy access, and diversity. Internet games have higher event frequencies that in turn lead to instant reinforcement and the ability to forget about losses (Griffiths and Barnes 2008).

When it comes to feeling trust, confidence, protection, and security while gambling online (i.e., because of the opportunities to self-exclude, the ability to set time and money limits, etc.), almost all PGON had higher mean scores and were more statistically significant than PGOF. Gamblers appear to have become less suspicious about the online gambling mode. The most highly rated variables that revealed a stronger bond between a pathological gambler and the Internet medium were having privacy, more information (probabilities, rules, dangers), historical information (previous bets, moves/hands), time and money control, self-exclusion, protection from criminality, and professional help. The relationship of gamblers in online modes must be used by health professionals to implement more online interventions (i.e., online treatment, online helplines, online information, and online self-help groups).

The importance of gambling for money (83.6% for PGOF and 76.3% for PGON) showed how important money became relative to the online gambling mode. New technologies have brought convenient accessibility and individuals appear to have more trust concerning online security and money transfers in recent years. Money and the attempt to win it is a fundamental factor in problem gambling, especially chasing losses and all its related the consequences. Nevertheless, PGON felt they had spent less money, won less money, and had fewer significant wins/rewards during gambling sessions, even though the figures are rather similar when compared to PGOF. Such a finding may show how money spent online seems of lesser value to online gamblers. In addition to socialization, research suggest that youth view gambling as an activity to win money (Wood and Griffiths 2004), and have beliefs that gambling can be a lucrative activity (Delfabbro et al. 2006) perhaps indicating that adolescents overestimate their chances of winning (Wood and Griffiths 2002).

PGON spent more time gambling in terms of number of days per week (PGON = 5.14 vs. PGOF = 3.33) but were almost equal in terms of hours per day and hours per weekend day. PGON were also more likely than PGOF to believe they could gamble for longer periods of time without it causing problems. In general, this perception of time and life priorities may be seen as another cognitive distortion that affects social, marital, professional, and/or academic goals. The lack of awareness or the selective memory in relation to time could be due to, among others: age, family dysfunctionality, comorbidities, denial, poor coping mechanisms, as well as, of course, familiarity and easy access to a PC and the Internet. Structural changes in games could perhaps help online pathological gamblers to overcome immersive feelings and lead to healthier gambling with the known online tracking strategies (i.e., amount of time and money spent, pop ups, self-exclusion, etc.) that may work even better online than offline.

PGON also felt they increased the money and time they spent last year, as they also gambled more during all periods of the day except for the night time (1 am to 9 am). This “flow” or feeling of time passing very fast while gambling during the daytime combined with the fact that they gamble mainly from their home computer, laptop in several places, and mobile phone, may indicate again a very strong, systematic, omnipresent, and continuous relationship with gambling. This is reinforced by the results, which showed that only 36.8% of PGON gamble online with other players, meaning that the majority prefer to gamble alone. In general, situational factors were more important for PGON than PGOF and individual factors were more important to PGOF than PGON. Significant differences exist between PGOF and PGON, but also significant similarities in relation to harm-related characteristics that may enable/facilitate gambling problems. PGON were more attracted to the situational characteristics that represent the core issues of the internet’s attractiveness, while PGOF were more influenced by individual characteristics, although several of these were not statistically different to PGON.

Internet gambling problems may become a phase in the risk factor continuum for both offline and online vulnerable gamblers. Online vulnerable gamblers reach the pathological gambling phase by 30 years old and PGOF by 40 years old. PGOF may be starting to bet more in an online mode and it may be that they accelerate their problems by also gambling online with all its attractive factors. Possibly most offline and online pathological gamblers will gamble more and more in both modes, becoming mixed-mode pathological gamblers. These mixed-mode gamblers had the highest rates of gambling involvement and higher problem gambling prevalence rates (Wardle et al. 2011). Considering that many of the gamblers in the present study gambled in mixed modes, future studies should further examine the relationship between different modes of gambling within individuals (i.e., gambling both online and offline). Ease of access, potential frequency of play, potential length of play, and the immersive qualities of the Internet medium itself may pose a unique set of challenges, along with a unique set of opportunities, for crafting effective awareness and prevention programs for the problem gambling of Internet gamblers (Wood and Williams 2007).

There are a number of implications arising from the findings of the present study at different levels. There is a need to (i) intensify treatment and prevention programs for online gamblers with particular focus concerning age, online culture, communication types, and psychotherapeutic setting (including families); (ii) develop different treatment and prevention strategies according to different types of online gambling (poker, sport betting, video gaming, etc.); (iii) educate and inform regulators and gambling promoters about the impact of varying situational and structural features including marketing, responsible gambling tools, types of messaging, near misses, in-play betting, etc., (iv) incorporate adequate and truthful information dissemination to gamblers, their families, local communities, and the media; and (v) establish new teaching programs concerning online gambling issues, to healthcare and treatment professionals, those that work in the online gambling industry, regulators, policymakers, and other public services where gambling can impact.

There are of course limitations to the present study. This was a non-representative study with a selective sample and simultaneously a self-report study with all its inherent limitations and biases (e.g., recall biases, social desirability, etc.). Some qualitative response options may also have provided essential information not provided by the forced options in the survey. Future studies should also examine at-risk gamblers more closely. It would also be useful to examine more closely the different motivations to gamble and types of gambling among mixed mode gamblers (i.e., those that gamble both online and offline) compared to gamblers that only gambled via a single medium (i.e., online only or offline only). Very little national data or scientific studies on adult problem gambling exist in Portugal prior to this study, and almost nothing on online gambling. The information presented here concerning sociodemographic variables, types of gambling behavior and individual/situational risk factors concerning offline and online pathological gamblers, is of great existential value. It is hoped that the findings of present study will stimulate further investigation into gambling in Portugal.
